# Supplementary material for: Searching for Metabolic Markers of Stroke in Human Plasma via NMR Analysis
Source: Int J Mol Sci. 2023 Nov 10;24(22):16173. doi: 10.3390/ijms242216173 (PMC10671802; doi:10.3390/ijms242216173)
Supplement: Supplementary file 1 [file ijms-24-16173-s001.zip › Supplementary Figure S1.pdf]

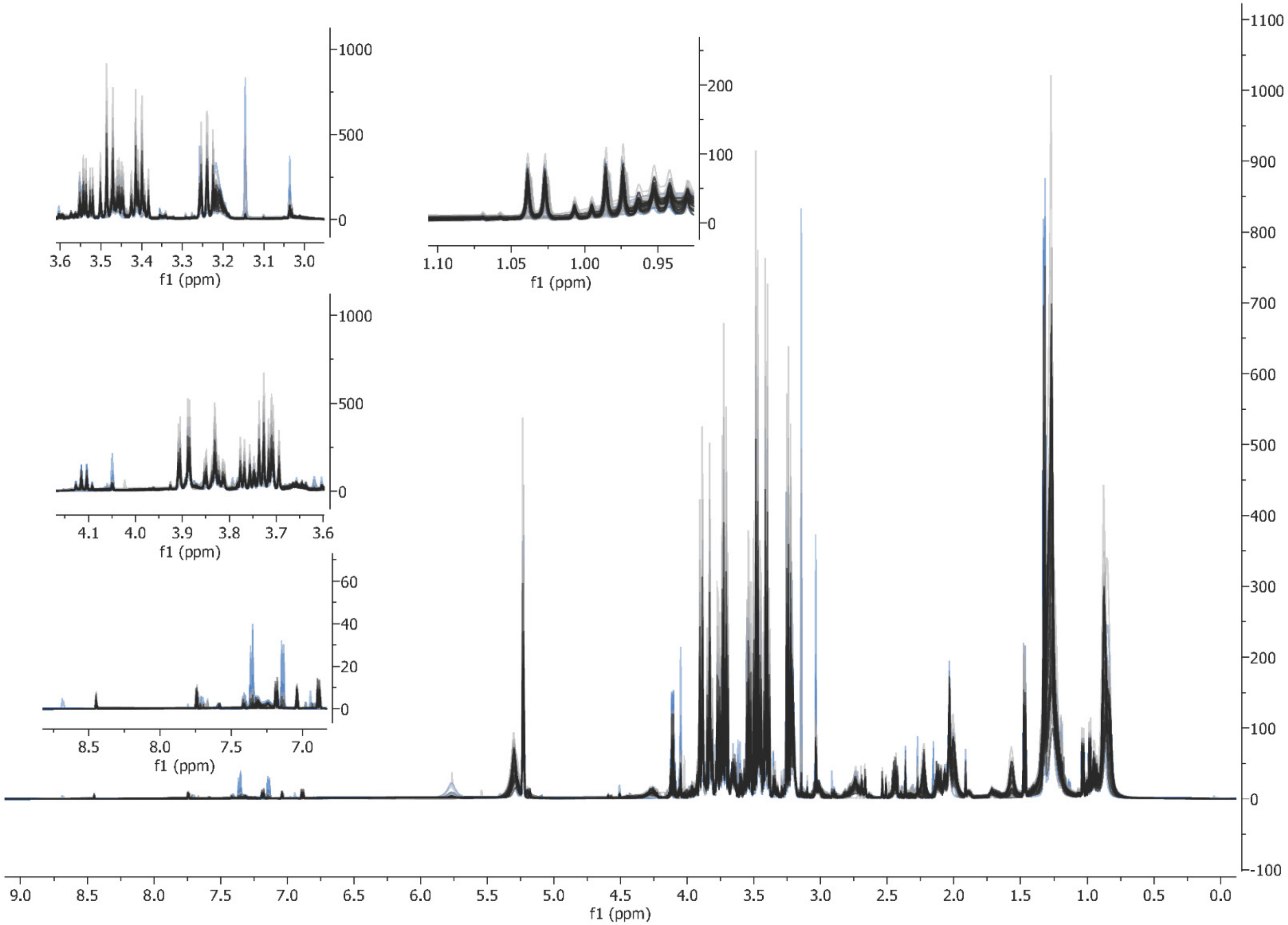

Figure S1 – Overlapping spectra of all plasma samples by groups. Overlapped 600MHz 1H-NMR CPMG plasma spectra of individuals from low stroke risk group (blue), moderate stroke risk group (grey) and high stroke risk group (black).
