# Supplementary material for: Searching for Metabolic Markers of Stroke in Human Plasma via NMR Analysis
Source: Int J Mol Sci. 2023 Nov 10;24(22):16173. doi: 10.3390/ijms242216173 (PMC10671802; doi:10.3390/ijms242216173)
Supplement: Supplementary file 1 [file ijms-24-16173-s001.zip › Supplementary Table S3.pdf]

Table S3 – Detailed comparative analysis of the metabolomic studies reporting the metabolites isoleucine, asparagine, creatinine, and formate as possible stroke biomarkers. ▼,▲ = increase or decrease, respectively, of metabolite levels.

Study type	Stroke type	Method	People (n)	Place	Age (years)	Sample	Metabolites		Authors
Cohort study	Ischemic & haemorrhagic stroke risk	NMR	197	Long-term care facilities	80-91	Plasma	▼▲ Isoleucine<br>▲ Asparagine<br>▲ Formate<br>▲ Creatinine	▲ Dimethyl sulfone<br>▲ Unknown 1<br>▲ Unknown 3	Present study
Prospective cohort study	Thrombotic	UPLC-MS/MS	400	Hospital	42-53	Serum	▲ L-tryptophan<br>▲ 3-methoxytyramine<br>▲ Methionine<br>▲ Homocysteine sulfinic acid<br>▲ Cysteine	▲ Isoleucine<br>▲ Carnitine<br>▲ Arginine<br>▲ Linoleic acid<br>▲ Sphingosine	Khan et. al (13)
Longitudinal cohort study	Ischemic	LC-MS	20	Hospital	57	Serum	▲ Asparagine<br>▲ Tyrosine	▲ Xylose	Sidorov et. al (20)
Cohort study	Ischemic	UPLC-MS/MS	216	Hospital	63-65	Serum	▲ Ornithine,<br>▲ Asparagine<br>▲ Valine<br>▲ Cysteine	▲ Glutamine<br>▲ Phenylalanine<br>▲ Tyrosine<br>▼ Citrulline	Tao et. al (22)
Cohort study	Cerebral infarction	NMR	58	Hospital	68-69	Plasma	▲ Lactate<br>▲ Pyruvate<br>▲ Glycolate	▲ Formate<br>▼ Glutamine<br>▼ Methanol	Jung et. al (23)
Cohort study	Ischemic	LC-MS/MS	84	Hospital	≥18	Plasma	▼ Valine<br>▼ Leucine	▼ Isoleucine	Kimberly et. al (27)
Longitudinal cohort study	Ischemic & haemorrhagic	LC-MS/MS	970	Hospital	67-70	Plasma	▲ Valine<br>▲ Leucine	▲ Isoleucine	Ruiz-Canela et al. (36)

Study type	Stroke type	Method	People (n)	Place	Age (years)	Sample	Metabolites		Authors
Cohort study	Ischemic and transient ischemic attack	UHPLC - MS/MS	60	Hospital	66-67	Serum	▲ Carnitine<br>▲ Creatinine<br>▲ Proline<br>▲ N-acetylneuraminic acid<br>▲ Hypoxanthine,<br>▲ Uric acid<br>▲ Tyrosine<br>▲ Kynurenine<br>▲ Phenylalanine	▲ Sphingosine-1-phosphate<br>▲ Palmitoylcarnitine<br>▼ Citric acid<br>▼ Valine<br>▼ Isoleucine<br>▼ Tryptophan<br>▼ LysoPCs	Liu et al. (38)
Prospective cohort study	Ischemic and haemorrhagic stroke risk	NMR	799	Hospital	54-55	Plasma	▼ Valine<br>▼ Leucine	▼ Isoleucine	Tobias et al. (39)
