# Supplementary material for: Searching for Metabolic Markers of Stroke in Human Plasma via NMR Analysis
Source: Int J Mol Sci. 2023 Nov 10;24(22):16173. doi: 10.3390/ijms242216173 (PMC10671802; doi:10.3390/ijms242216173)
Supplement: Supplementary file 1 [file ijms-24-16173-s001.zip › Supplementary Tables S1 and S2.pdf]

Supplementary material

Table S1 – Assignments of chemical shifts of metabolites from 600MHz ^{1}H-NMR CPMG spectra. Peaks multiplicities are represented by singlet (s), doublet (d), triplet (t), multiplet (m) and double doublet (dd).

Metabolites	Chemical Shift (ppm)	Multiplicity
Isoleucine	1.00	d
Leucine	0.94	t
Valine	0.99	dd
3-Hydroxybutyric acid	1.19	d
Lactic Acid	1.32; 4.10	d; m
Alanine	1.47	d
Lysine	1.90; 3.01	m; t
Acetate	1.91	s
Proline	2.02	m
Glycerol	2.03; 3.61	s, m
Glutamine	2.12; 2.42	m; m
Acetone	2.22	s
Glutamate	2.32	m
Pyruvate	2.36	s
Citrate	2.56; 2.66	d; d
Asparagine	2.89	m
Creatine	3.02; 3.92	s; s
Creatinine	3.03; 4.04	s; s
Unknown 1	3.1	s
Dimethyl sulfone	3.14	s
Unknown 2	3.22	s
Glucose	3.23; 3.40; 3.49; 3.53;3.88; 5.23	m; m; m; m; dd; d
Betaine	3.26	s
Glycine	3.55	s
Threonine	3.60	d
Unknown 3	3.75	s
Unknown 4	3.79	s
Urea	5.78	s
Tyrosine	6.90; 7.19	d; d
Histidine	7.04; 7.73	s; s
Formate	8.44	s

Table S2 – Relative concentrations of the 31 metabolites/signals identified in plasma samples from elderly people belonging to the study groups, namely low stroke risk (LSR, control, n=85), moderate stroke risk (MSR, n=94), and high stroke risk (HSR, n=18). Data are expressed as "Mean ± s.e.m"." Statistical significance was analysed with Kruskal-Wallis test (KWT) and One-way ANOVA test (OWAT) depending on the homogeneity of variance of the data (*$p < 0.05$).

Metabolites	LSR	MSR	HSR	P-value	Statistical test
Isoleucine	176.22 ± 3.84	163.93 ± 3.62	178.38 ± 8.06	0.040*	OWAT
Leucine	642.28 ± 14.12	617.80 ± 13.35	640.61 ± 24.66	0.415	OWAT
Valine	515.69 ± 11.22	490.17 ± 9.35	531.01 ± 19.33	0.101	OWAT
3-Hydroxybutyric acid	154.99 ± 8.65	143.36 ± 7.37	164.99 ± 17.38	0.402	OWAT
Lactic Acid	2939.75 ± 92.55	3208.44 ± 97.14	3275.79 ± 270.96	0.115	OWAT

Alanine	1146.50 ± 33.27	1154.51 ± 31.62	1233.97 ± 57.03	0.522	OWAT
Lysine	228.11 ± 3.73	217.36 ± 4.02	227.89 ± 5.19	0.113	OWAT
Acetate	61.13 ± 3.11	64.88 ± 3.07	58.90 ± 7.28	0.588	OWAT
Proline	502.25 ± 20.61	521.49 ± 22.80	652.97 ± 80.61	0.322	KWT
Glycerol	1906.52 ± 36.84	1893.19 ± 28.41	1910.53 ± 56.01	0.947	OWAT
Glutamine	1430.14 ± 22.69	1468.13 ± 15.59	1507.44 ± 51.46	0.214	KWT
Acetone	34.05 ± 1.67	34.83 ± 1.61	46.79 ± 9.79	0.355	KWT
Glutamate	311.37 ± 6.60	305.75 ± 6.42	307.65 ± 10.73	0.823	OWAT
Pyruvate	138.58 ± 5.22	138.42 ± 5.23	137.56 ± 11.59	0.997	OWAT
Citrate	256.95 ± 5.49	268.38 ± 4.74	301.90 24.07	0.164	KWT
Asparagine	122.46 ± 2.80	115.33 ± 2.26	137.80 ±7.56	0.001*	OWAT
Creatine	62.29 ± 3.79	70.83 ± 3.54	62.88 ± 7.91	0.234	OWAT
Creatinine	217.97 ± 7.34	208.24 ± 6.81	258.53 ± 20.30	0.016*	OWAT
Unknown 1	38.69 ± 2.23	41.54 ± 2.37	54.47 ± 6.64	0.024*	OWAT
Dimethyl sulfone	31.24 ± 1.77	30.50 ± 1.92	44.82 ±6.33	0.009*	OWAT
Unknown 2	161.54 ±7.86	173.07 ± 7.13	198.33 ±13.05	0.111	OWAT
Glucose	2320.98 ± 39.96	2282.79 ± 32.33	2473.35 ± 117.18	0.107	OWAT
Betaine	181.81 ± 10.73	170.77 ± 11.26	204.03 ±33.93	0.454	OWAT
Glycine	428.66 ± 13.00	415.28 ± 13.85	391.43 ± 27.95	0.485	OWAT
Threonine	189.23 ± 4.67	183.01 ± 3.89	201.76 ±8.62	0.162	OWAT
Unknown 3	28.31 ± 1.13	29.16 ± 1.01	34.87 ± 2.27	0.036*	OWAT
Unknown 4	371.14 ± 16.18	345.65 ± 15.10	427.76 ± 32.55	0.082	OWAT
Urea	176.80 ± 4.03	183.22 ± 4.81	212.66 ± 8.68	0.004*	OWAT
Tyrosine	106.17 ± 2.25	103.74 ± 1.99	112.70 ± 6.33	0.228	OWAT
Histidine	67.77 ± 1.01	66.93 ± 1.10	70.99 ± 2.08	0.273	OWAT
Formate	11.16 ± 0.26	12.05 ± 0.35	13.65 ± 0.91	0.031*	KWT
